# Drug-eluting Ti wires with titania nanotube arrays for bone fixation and reduced bone infection

**DOI:** 10.1186/1556-276X-6-571

**Published:** 2011-10-31

**Authors:** Karan Gulati, Moom Sinn Aw, Dusan Losic

**Affiliations:** 1 Ian Wark Research Institute, University of South Australia, Mawson Lakes Boulevard, Mawson Lakes, Adelaide, South Australia, 5095, Australia

**Keywords:** Kirschner wires, titanium wires, titania nanotubes, bone fixation, bone infection, gentamicin

## Abstract

Current bone fixation technology which uses stainless steel wires known as Kirschner wires for fracture fixing often causes infection and reduced skeletal load resulting in implant failure. Creating new wires with drug-eluting properties to locally deliver drugs is an appealing approach to address some of these problems. This study presents the use of titanium [Ti] wires with titania nanotube [TNT] arrays formed with a drug delivery capability to design alternative bone fixation tools for orthopaedic applications. A titania layer with an array of nanotube structures was synthesised on the surface of a Ti wire by electrochemical anodisation and loaded with antibiotic (gentamicin) used as a model of bone anti-bacterial drug. Successful fabrication of TNT structures with pore diameters of approximately 170 nm and length of 70 μm is demonstrated for the first time in the form of wires. The drug release characteristics of TNT-Ti wires were evaluated, showing a two-phase release, with a burst release (37%) and a slow release with zero-order kinetics over 11 days. These results confirmed our system's ability to be applied as a drug-eluting tool for orthopaedic applications. The established biocompatibility of TNT structures, closer modulus of elasticity to natural bones and possible inclusion of desired drugs, proteins or growth factors make this system a promising alternative to replace conventional bone implants to prevent bone infection and to be used for targeted treatment of bone cancer, osteomyelitis and other orthopaedic diseases.

## Introduction

Kirschner wires [K-wires] are smooth stainless steel pins that have been widely used for temporary and definitive bone fixation, especially if the fracture fragments are small, e.g. wrist fractures and hand injuries [1]. K-wires are generally passed through the skin, then transversely through the bone and out of the other side of the limb. This results in a potential passage for bacteria from the skin to migrate into the bone and cause an infection, referred to as pin tract infection [1]. Such infections are generally caused by *Staphylococcus aureus *and *Staphylococcus epidermidis *which can adhere to the implant surface forming biofilms [2,3]. These biofilms impair treatment and bone tissue healing as bacteria are protected from the antibiotics [4]. Implant-associated infection is often treated with systemic administration of antibiotics and pin removal which compromises patient compliance and leaves fractures unfixed. If left unattended and unmanaged, this infection can lead to severe complexities like osteomyelitis, septic arthritis and similar problems [5]. Also, it has been cited that with the use of external bone fixators, the infection rate can be as high as 33% [6]. A possible solution to these problems is the coating of pins with antibiotics or to modify the implant surface to prevent such bacterial growth and infection [7,8]. Another strategy is to replace such bone fixation stainless steel wires with another material where titanium, with regard to its proven biocompatibility, osseointegrating and superior mechanical properties, is an excellent choice [9].

Titania nanotube [TNT] arrays generated on a Ti surface by electrochemical anodisation have been extensively explored in the past several years for drug delivery systems, cell growth, biosensors and tissue engineering [10-13]. TNTs fabricated on a Ti implant surface can serve as carriers of drugs, proteins or growth factors for their localised delivery from an implant surface, which aid in reducing the incidence of infection or impaired bone healing [14-16]. Studies have established the capability of TNTs for local delivery of different therapeutics including water insoluble drugs, antibiotics and sensitive drugs such as proteins from the implant surface at the site of implantation [11,14-17]. It was proven that the surface of antibiotic-loaded TNTs is capable of reducing bacterial adhesion whilst retaining the normal osteoblast adhesion and differentiation [18-20]. Studies from our group demonstrated several strategies to extend drug release from TNT implants which include controlling of nanotube structures, their surface modification, polymer coating and loading drugs into nanocarriers (polymer micelles) [21-23]. By coating TNT with biocompatible polymers such as poly(lactic-*co*-glycolytic acid) [PLGA] and chitosan, an extended release of water insoluble drugs up to more than 30 days and an improved adhesion proliferation of osteoblast cells were achieved [24].

Another advantage of using Ti is its lower modulus of elasticity, which matches more closely to that of the bone as compared with that of stainless steel K-wires. Hence, the skeletal load can be more evenly shared between the bone and the implant, resulting in a lower incidence of bone degradation due to stress shielding. Also, a TNT layer has a much closer elastic modulus to that of natural bones, and hence, it is expected to have a better biomechanical compatibility as compared with other implant materials [25]. Thus, Ti with a TNT layer has a great potential promise in aiding enhanced bone healing and implant survival with minimised infection problems.

**Figure 1:**
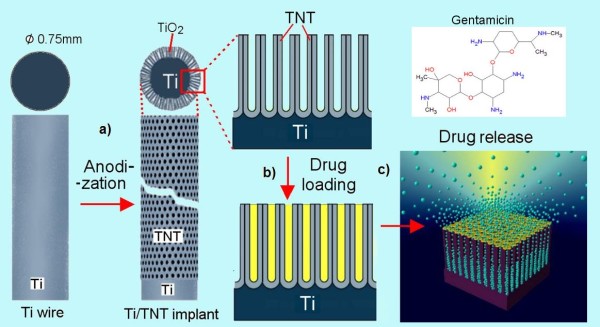
**Scheme of titania nanotubes fabricated on Ti wire as a bone implant**. (**a**) TNT layer formed on a cleaned Ti wire using electrochemical anodisation, (**b**) the loading drugs inside TNT structures and (**c**) the release of drug molecules from TNTs immersed in phosphate buffer.

In this study, we investigated the feasibility of titanium wire with TNT layers as a drug carrier for local antibiotic therapy and extended drug release characteristics. A schematic of TNT-Ti wire implants is shown in Figure 1. We propose this system using Ti wire with drug-eluting ability as an improved bone fixative in comparison with the current K-wire technique, which could promote bone healing and prevent infection incidence for extended durations. Gentamicin, a common aminoglycoside antibiotic widely used for oral therapy associated with bacterial infection due to the implant, was selected as our model to explore the release characteristics of our system [26]. In comparison with conventional drug administration, this approach provides several advantages by employing the drug release from the bone fixative surface, directly to the infected area around the implant, with enhanced anti-bacterial activity to reduce chances of infection.

## Experiment

### Materials

Titanium wire (99.7%) with a diameter of 0.75 mm was supplied by Alfa Aesar (MA, USA). Ethylene glycol, ammonium fluoride [NH_4_F] and gentamicin sulfate were obtained from Sigma-Aldrich (New South Wales, Australia). High purity Milli-Q water (Millipore Co., Billerica, MA, USA), ultra-pure grade (18.2 MΩ) and sieved through a 0.22- μm filter, was used.

### Fabrication of TNT arrays on Ti wires

The titanium wire was cut into a length of 2.5 cm, mechanically polished and cleaned by sonication in acetone for 30 min prior to anodisation. Two anodisation steps were performed using a specially designed electrochemical cell and computer-controlled power supply (Agilent Technologies Inc.) and a previously described procedure [27,28]. In the first anodisation step, a constant voltage of 100 V was applied for 1 h in ammonium fluoride/ethylene glycol electrolyte (3% water and 0.3% NH_4_F) at a room temperature of 20°C. The resultant layer of anodic TNT layer was removed (by sonication in methanol), leaving the nanotextured titanium surface for the second anodisation. The second anodisation step to make the final TNT layer on Ti wire was performed using the same conditions. The voltage-current, voltage-time and current-time signals were adjusted and continuously recorded during the anodisation process by a software (Labview, National Instruments, Austin, TX, USA).

### Structural characterisations

The structural characterisation of the prepared TNT/Ti wires before/after drug loading and drug release was performed using a field emission scanning electron microscope [SEM] (Philips XL 30, SEMTech Solutions, Inc., North Billerica, MA, USA). The samples were cut into small (approximately 5 mm) pieces, mounted on a holder with a double-sided conductive tape and coated with a layer of platinum 3 to 5 nm thick. Images with a range of scan sizes at normal incidence and at a 30° angle were acquired from the top, the bottom surface and the cross-sections.

### Drug loading

A drug solution of 1% (*w*/*v*) gentamicin sulfate in water was prepared. Ti wires with a TNT surface were cleaned using deionised water and dried in nitrogen; 100 μl of the drug solution was pipetted onto the nanotube surface and allowed to dry in air. After drying, the TNT surface was using a soft tissue in order to remove excess drug accumulated on the surface. The wire was rotated after each step to ensure that the drug was loaded into nanotubes all around the wire. Loading, drying and wiping steps were repeated 20 times in order to load a substantial amount of drug into the nanotubes.

### Quantitative analysis of drug loading

To determine the amount of drug loaded in the nanotubes, thermo-gravimetric analysis [TGA] was performed. In order to find the correct range of the drug decomposition, 20 to 25 mg of drug was loaded into the platinum pan in TGA and heated in the burning furnace from 20°C to 800°C, and its characteristic peak was obtained. Later, the drug-loaded TNTs were characterised, and the peak of the drug was identified in order to calculate the correct amount of drug present.

### Drug release characterisation

Drug release from the drug-loaded TNT-Ti wire samples was investigated by their immersion in 5 ml phosphate-buffered saline [PBS], where the amount of released drug was measured using ultraviolet-visible [UV-Vis] spectroscopy, as described previously [23]. Measurements were taken at short intervals during the first 6 h to monitor the initial burst release, followed by testing every 24 h to observe the delayed release until the entire drug amount was released into the surrounding PBS. Absorbance was measured at 290 nm, and the corresponding drug concentration was calculated based on the calibration curve obtained for the drug. Ultimately, the release profiles of each experimental set were expressed for burst and delayed releases in a plot with release percentage vs. time. Drug release percentage (weight percentage) is calculated from the amount of drug released into the buffer solution, divided by the total amount of drug (weight) released at the end of the release (determined by UV-Vis spectrophotometer) and multiplied by 100.

## Results and discussion

**Figure 2:**
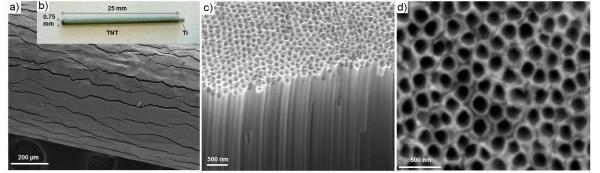
**SEM images of TNT grown on Ti wire using the anodisation technique**. (**a**) The top surface showing cracks, (**b**) the entire structure showing TNT on Ti wire with dimensions, (**c**) the cross-section showing array of TNTs and (**d**) the hollow nanotubes.

The morphology of the prepared TNT-Ti wires was characterised by SEM and is summarised in Figure 2. A low-resolution SEM image of the wire surface is presented in Figure 2a and an image of the whole TNT-Ti wire (25 mm) is presented in Figure 2b, confirming the radial growth of TNT film on the Ti wire. The thickness of the TNT layer was about 72 μm, which was controlled by selecting the appropriate voltage (100 V) and anodisation time (1 h). The formed TNT layer showed numerous cracks with a width of 1.8 μm and 1 to 2 mm long, across the wire length. The cracks were found on the entire length of the TNT layer that extend to the bottom and reach the Ti wire. These fractures of TNT film were created as results of radial growth and mechanical stress caused by volume extension of the formed TNT oxide layer on the circular surface of Ti wire and were not observed on planar Ti surface [28,29]. When thinner TNT layers were prepared, the width of these fractures was considerably smaller. A high-resolution SEM image of the top surface and cross-sections of the TNT layer shows a vertically aligned and densely packed array of nanotubes across the entire structure (Figure 2c, d). SEM images of the top nanotube surface (Figure 2c) show pores with diameters of 170 ± 10 nm. The end of the tubes at the Ti interface is closed with a barrier layer and has considerably reduced pore diameters (data not shown). In this study, TNTs synthesised on curved and circular surfaces has been  reported for the first time and instead of observed fractures, the TNT film was  found to be mechanically stable and hard to remove from the Ti wire. Also these  micrometer range fractures/gaps are beneficial for the growth of bone cells and osseointegration of implants.

**Figure 3:**
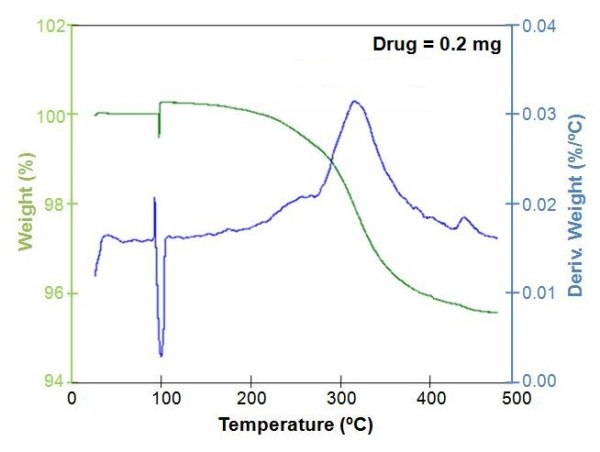
**TGA plot showing the amount of drug (gentamicin) loaded inside TNTs**.

To prove the drug-loading and drug-eluting abilities of our system, gentamicin, a common antibiotic, was selected as a model. TGA studies (Figure 3) confirmed the successful loading of this drug inside the TNT with a loading amount of around 0.2 mg (or 200 μg) for a 2.5-cm wire length. For this study, TNTs with larger pore diameters and greater lengths were prepared, in order to maximise their loading capacity. The surface area and total volume of nanotube reservoirs in a TNT layer are enormous, and the amount of loaded drug has the capacity to provide a very high local concentration of antibiotics which is essential to suppress bacterial infection. More importantly, drug-loading capacity can be precisely tuned by controlling nanotube structures by the anodisation condition and by the size of the implant (Ti wire). This is an important feature of TNT-Ti implants to meet specific requirements, depending on the drug, implant size, bone and specific clinical conditions. Also, the system is generic such that different types of drugs, proteins or growth factors (including their mixtures) could be loaded, thereby providing the ability to design TNT-Ti wire implants with multiple drug release and complex bone therapies, including bone infections and metastatic bone cancer.

**Figure 4:**
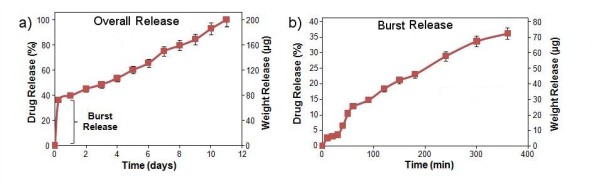
**Drug release graph of gentamicin from TNT-Ti wire**. (**a**) Overall release and (**b**) burst release (corresponding to the first 6 h of fast diffusion of drug).

Drug release profiles of gentamicin loaded into the TNT-Ti wire are presented in Figure 4 showing both the fast (burst) phase and overall releases. The release characteristics are listed in Table 1, which shows the release efficiency (percentage of drug release) at various time intervals. The drug release kinetics can be described in two phases, with burst release of the drug released in the first 6 h when 37% of drug is released, followed by slow release over the following 11 days. This fast initial release accounts for the fast diffusion of the loosely bound drug molecules at the top part of the TNT, due to a high concentration gradient between the drug interface at the TNT layers and the bulk PBS solution. The amount of drug released during this period is approximately 72 μg and is appropriate to have a high local concentration of antibiotic during the initial few hours after the orthopaedic surgery to prevent bone infections.

**Table 1:** The release characteristics of gentamicin from TNT-Ti

Time	1 h	6 h	1 day	3 days	7 days	11 days
Drug release (%)	12.7 ± 1.2	36.2 ± 0.8	39.6 ± 0.5	48.5 ± 4.2	75.1 ± 13.6	100.0 ± 0.0
Weight release (μg)	25.4 ± 3.3	72.4 ± 1.4	79.2 ± 0.9	97.0 ± 5.8	150.2 ± 24.1	200 ± 0.1

The release characteristics of gentamicin from TNT-Ti (mean ± SD, *n *= 3) showing drug release (%) and weight release (μg) at different time intervals determined by UV-Vis spectrophotometry. The total amount of loaded drug was 200 μg determined by TGA.

In the second phase, different kinetics of drug release is observed from TNT-Ti with a very slow and linearly increased cumulative release over 11 days when no drug is detected inside the TNTs (Figure 4a). The release kinetics of this phase is controlled by a diffusion process from the deep nanotube structures (70 μm). For this stage, it is suggested that the gentamicin release mechanism is due to the diffusive transport through the ordered array of TNT since it is an insoluble matrix. Considering the high surface area and long capillary-like structures of TNTs, diffusion of the gentamicin drug to PBS can be described as a surface-dependent phenomenon. The TNT surface is negatively charged, and because the prominent chemical groups of gentamicin is aminoglycoside with amino groups (Figure 1) which are positively charged, an electrostatic interaction with the TNT surface could also have an influence on a long release observed on this drug.

The best fitting model for the gentamicin release data was observed using the Higuchi and zero-order releases, which describe drug release from an insoluble matrix [30]. The square root of a time-dependent process is based on Fickian's diffusion law where diffusion-controlled release rate of drug molecules decreases as a function of time due to a reduction in the concentration gradient. The pharmaceutical dosage following the zero-order profile is the ideal method of drug release, providing the same amount of drug per unit of time. Our results confirmed that drug release into the local environment during this time was constant with a value of approximately 12 μg every day. By controlling the dimensions of TNT structures (diameter and length), this local concentration can be controlled and tuned to fit into an optimal therapeutic window for the treatment of bone infection by antibiotic. The general approach for antibiotic treatments through implantable devices requires a large drug loading and constant release over extended periods (e.g. weeks). To address this problem, we recently introduced several approaches to considerably extend drug release from TNT using polymer micelles and polymer coatings (plasma polymers, chitosan, PLGA) [23,24]. These approaches can be applied here to achieve a long and sustained release of antibiotic with a desired concentration and zero-order kinetics over more than 4 weeks.

## Conclusions

In our study, we report a new approach of preparing drug-eluting Ti implants in the form of Ti wires with a layer of TNT arrays fabricated as a bone fixative tool or an orthopaedic implant. A simple and cost-effective electrochemical technique was used for the synthesis of TNT arrays on Ti wire, followed by the loading of a common antibiotic drug, gentamicin. The drug loading and release of the model antibiotic drug (gentamicin) were characterised to reveal drug-eluting characteristics of our proposed implant. This system with TNT on Ti wires can be applied as a bone fixative tool, an implant or for complex bone ailments (for drug elution inside bones). The wire can easily be inserted inside the bones and could potentially open up new possibilities for enhanced bone fixation/repair and targeted treatment of bone cancer, osteomyelitis and other related orthopaedic diseases.

## Competing interests

The authors declare that they have no competing interests.

## Authors' contributions

KG carried out all experimental works including the preparation of TNT-Ti, SEM characterisation, drug loading and release studies and the writing of the manuscript draft. MSA was involved in the evaluation and discussion of release kinetics. DL provided knowledge and supervision support for this study and wrote the final version of the paper. All the authors read and approved the final manuscript.

## References

[B1] Mahan J Seligson D Henry SL Hynes P Dobbins J Factors in pin tract infections Orthopedics 1991 14 305 308 2020629

[B2] Mahan J Seligson D Henry SL Hynes P Dobbins J In vitro and in vivo comparative colonization of *Staphylococcus aureus *and *Staphylococcus epidermidis *on orthopaedic implant materials Biomaterials 1989 10 325 328 10.1016/0142-9612(89)90073-2 2765629

[B3] von Eiff C Proctor RA Peters G Coagulase-negative staphylococci. Pathogens have major role in nosocomial infections Postgrad Med 2001 110 63 70 11675983

[B4] Hoyle BD Costerton JW Bacterial resistance to antibiotics: the role of biofilms Prog Drug Res 1991 37 91 105 1763187 10.1007/978-3-0348-7139-6_2

[B5] Birdsall PD Milne DD Toxic shock syndrome due to percutaneous Kirschner wires Injury 1999 30 509 510 10.1016/S0020-1383(99)00142-4 10707221

[B6] Hargreaves DG Drew SJ Eckersley R Kirschner wire pin tract infection rates: a randomized controlled trial between percutaneous and buried wires J Hand Surg-Brit Eur 2004 29 4 374 376 10.1016/j.jhsb.2004.03.003 15234503

[B7] Collinge CA Goll G Seligson D Easley KJ Pin tract infections: silver vs uncoated pins Orthopedics 1994 17 445 448 8036188 10.3928/0147-7447-19940501-11

[B8] Wassall MA Santin M Isalberti C Cannas M Denyer SP Adhesion of bacteria to stainless steel and silver-coated orthopaedic external fixation pins Biomed Mat Res 1997 36 325 330 10.1002/(SICI)1097-4636(19970905)36:3<325::AID-JBM7>3.0.CO;2-G 9260103

[B9] Liu H Webster TJ Nanomedicine for implants: a review of studies and necessary experimental tools Biomaterials 2007 28 354 369 10.1016/j.biomaterials.2006.08.049 21898921

[B10] Ghicov A Schmuki P Self-ordering electrochemistry: a review on growth and functionality of TiO(2) nanotubes and other self-aligned MO(x) structures Chem Commun 2009 20 2791 2808 10.1039/b822726h 19436878

[B11] Losic D Simovic S Self-ordered nanopore and nanotube platforms for drug delivery applications Expert Opin Drug Deliv 2009 6 1363 1380 10.1517/17425240903300857 19860534

[B12] Oh S Daraio C Chen LH Pisanic TR Fiñones RR Jin S Significantly accelerated osteoblast cell growth on aligned TiO_2 _nanotubes J Biomed Mater Res A 2006 78 1 97 103 16602089 10.1002/jbm.a.30722

[B13] Park J Bauer S von der Mark K Schmuki P Nanosize and vitality: TiO_2 _nanotube diameter directs cell fate Nano Lett 2007 7 1686 91 10.1021/nl070678d 17503870

[B14] Popat KC Eltgroth M Latempa TJ Grimes CA Desai TA Decreased *Staphylococcus epidermis *adhesion and increased osteoblast functionality on antibiotic-loaded titania nanotubes Biomaterials 2007 28 4880 4888 10.1016/j.biomaterials.2007.07.037 17697708

[B15] Popat KC Eltgroth M LaTempa TJ Grimes CA Desai TA Titania nanotubes: a novel platform for drug-eluting coatings for medical implants Small 2007 3 1878 81 10.1002/smll.200700412 17935080

[B16] Song YY Schmidt-Stein F Bauer S Schmuki P Amphiphilic TiO_2 _nanotube arrays: an actively controllable drug delivery system J Am Chem Soc 2009 131 12 4230 4233 10.1021/ja810130h 19317500

[B17] Peng L Mendelsohn AD LaTempa TJ Yoriya S Grimes CA Desai TA Long-term small molecule and protein elution from TiO_2 _nanotubes Nano Lett 2009 9 1932 1936 10.1021/nl9001052 19323554

[B18] Aninwene G Yao C Webster TJ Enhanced osteoblast adhesion to drug coated anodized nanotubular titanium surfaces Int J Nanomedicine 2008 3 257 264 18686785 10.2147/ijn.s2552 PMC2527662

[B19] Burns K Yao C Webster TJ Increased chondrocyte adhesion on nanotubular anodized titanium J Biomed Mater Res A 2009 88 561 568 18306319 10.1002/jbm.a.31899

[B20] Oh S Brammer KS Li YSJ Teng D Engler AJ Chien S Jin S Stem cell fate dictated solely by altered nanotube dimension Proc Natl Acad Sci 2009 106 2130 2135 10.1073/pnas.0813200106 19179282 PMC2650120

[B21] Simovic S Losic D Vasilev K Controlled drug release from porous materials by plasma polymer deposition Chem Commun 2009 46 8 1317 1319 10.1039/b919840g 20449289

[B22] Losic D Velleman L Kant K Kumeria T Gulati K Shapter JG Beattie DA Simovic S Self-ordering electrochemistry: a simple approach for engineering nanopore and nanotube arrays for emerging applications Aust J Chem 2011 64 294 301 10.1071/CH10398

[B23] Aw MS Simovic S Addai-Mensah J Losic D Polymeric micelles in porous and nanotubular implants as a new system for extended delivery of poorly soluble drugs J Mater Chem 2011 21 20 7082 7089 10.1039/c0jm04307a

[B24] Gulati K Aw MS Ramakrishnan S Atkins GJ Findlay DM Losic D Polymer coating of titania nanotube arrays for improved drug elution and osteoblast adhesion Acta Biomater in press 10.1016/j.actbio.2011.09.004 21930254

[B25] Crawford GA Chawla N Das K Bose S Bandyopadhyay A Microstructure and deformation behavior of biocompatible TiO_2 _nanotubes on titanium substrate Acta Biomater 2007 3 359 367 10.1016/j.actbio.2006.08.004 17067860

[B26] Baro M Sánchez E Delgado A Perera A Évora C In vitro-in vivo characterization of gentamicin bone implants J Control Release 2002 83 3 353 364 10.1016/S0168-3659(02)00179-7 12387944

[B27] Vasilev K Poh Z Kant K Chan J Michelmore A Losic D Tailoring the surface functionalities of titania nanotube arrays Biomaterials 2010 31 3 532 540 10.1016/j.biomaterials.2009.09.074 19819014

[B28] Kant K Losic D A simple approach for synthesis of TiO_2 _nanotubes with through-hole morphology Phys Status Solidi-R R L 2009 3 5 139 141

[B29] Kant K Losic D Self-ordering electrochemical synthesis of TiO_2 _nanotube arrays: controlling the nanotube geometry and the growth rate International Journal of Nanoscience 2011 10 1 6 10.1142/S0219581X11007466

[B30] Reichal CR Lakshmi JB Ravi TK Studies on formulation and in vitro evaluation of Glimepiride floating tablets J Chem Pharm Res 2011 3 3 159 164

